# Predictive Variables of Adolescents’ Intention to Be Physically Active after Graduation. Is Gender a Conditioning Factor?

**DOI:** 10.3390/ijerph17124308

**Published:** 2020-06-16

**Authors:** María Huertas González-Serrano, Ana Gómez-Tafalla, Ferran Calabuig-Moreno

**Affiliations:** 1 Department of Teaching and Learning of Physical Education, Plastic and Music Education, Universidad Católica de Valencia, 46110 Valencia, Spain; mh.gonzalez@ucv.es; 2 Department of Physical Education and Sports, Faculty of Physical Activity and Sport Sciences, Universitat de València, 46010 Valencia, Spain; ana.maria.gomez@uv.es

**Keywords:** adolescence, gender, physical activity, the theory of planned behavior, physical self-concept, athletic identity

## Abstract

The acquisition of physical activity habits during adolescence is fundamental to guarantee its adherence throughout life. However, these levels decrease during this stage, with women experimenting a more significant decrease. This paper aims to analyze if there are significant differences in the variables related to the intention to be physically active between men and women and if there is a moderating effect of gender on the variables that predict this intention. The sample is composed of 256 adolescents, aged between 16 and 19 years, 55.50% of whom are men, and 44.50% women. The results show that there are statistically significant differences (*p* < 0.05), with boys showing higher scores in the intention, athletic identity, and in the strength, condition, and attractiveness. About the predictive variables of the intention, the gender moderates the relationships between the physical attractiveness, condition and strength with the perceived behavioral control (*pc*fmale-*pc*fmale = 0.44; −0.48 *p* < 0.001; 0.27, *p* < 0.05, respectively), and between the subjective norm and the intention to be physically active (*pc*male-female = 0.33, *p* < 0.01). These results highlight the importance of considering gender when designing specific policies for the promotion of physical-sports practice among adolescents to reduce the existing gender gap.

## 1. Introduction

Physical inactivity is a global pandemic [1], leading to the development of major diseases [2,3,4]. Sedentary lifestyles in children and adolescents continue to be a global public health problem [5,6], with levels of physical activity declining as children move into adolescence [7]. Since the adolescent stage is a crucial period for people to establish themselves as regular practitioners of physical activity throughout their lives, or to abandon it completely [8,9], this is worrying.

In addition to the fact that levels of physical inactivity during this stage of life remain very high worldwide, this is especially worrying in the case of girls [6]. Various researchers have shown that Spain is the country where they find the most significant differences in physical-sports practice concerning gender [10,11,12], with Spanish teenage girls being the least physically active in all of Europe [13,14,15]. Moreover, although the prevalence of insufficient physical activity significantly decreased between 2001 and 2016 for boys, there was no significant change for girls [6]. Thus, even though gender has been a well-studied variable about physical activity practice [16,17,18], there is a need for further study. Furthermore, despite this panorama, there is not enough scientific literature on the intention to be physically active with Spanish adolescents has been found [19,20], and even less, that they consider the gender [19].

According to [21], the differences generated between students of different genders appear gradually throughout their lives. At first young people do not show discriminatory judgments related to physical-sports practice. However, the thinking of adolescents is formed by the influence of socializing agents in their environment, which condition and transform the initial positive predisposition. Consequently, as time goes by, the approach of equality of children is being lost. For this reason, they have pointed out that the lower level of physical sports practice by women compared to men is due to cultural factors associated with gender. In some countries where resources have been invested to achieve gender equity, it is girls who practice more physical activity than boys [22]. Hence, it is important to study the moderating effect of gender on the variables that predict the intention to be physically active, due to the low levels of physical activity presented by the female adolescents compare to the male counter partners. Discovering the predictive variables of both male and female, the policies for promoting physical activity in adolescents, could be improved to increase the levels of adolescents ‘physical activity practice

Due to that situation, over the past three decades, researchers and educators have increasingly recognized the importance of gender education in sport [23]. A relationship between sport and masculinity continues to exist and is firmly embedded in the cognitive network of individuals [24]. In fact, [25] shows that men are more involved in sport than women since athletic identity is positively related to masculinity and negatively to femininity. Furthermore, some studies have shown that the moderating effect of the strength of attitude on intention depends on the sociodemographic characteristics of the samples, with different relationships contributing to the cognitive variables of TPB to explain the intention of the behavior [26,27]. Most behavioral health theories, such as physical activity, assume that a person’s behavior is the result of conscious intent [28], being the intention to be physically active a good predictor of the physical activity behavior.

The Theory of Planned Behavior (TPB) [29] has been widely used to predict behaviors. The underlying premise of the TPB [29], is based on the fact that behavior requires a certain amount of planning, which can predict the intention to adopt that It posits that individuals’ intention is shaped by their attitudes, subjective norms, and perceived behavioral control behavior. Also, numerous studies support the value of TPB for understanding physical activity in youth, and it is extensively used as a theoretical basis for explaining behavior in the physical activity and exercise domain [26,30,31,32]. Moreover, a recent study highlights that it has been one of the most used theories to predict physical activity behavior [33]. Therefore, adolescents who have a favorable attitude towards physical activity in a regular manner (attitude towards behavior), who perceive that people from their environment see physical activity practice as something desirable (subjective norm), and perceive that they have the control and capacity to practice physical activity regularly (perceived behavioral control), are more likely to declare strong intentions to engage in physical activity [34].

Along these lines, some studies highlight that a positive attitude towards physical activity practice is the predictor variable that carries the most weight in future intentions of physical activity practice [35]. In contrast, other studies present the perception of having the control and capacity to practice physical activity regularly, as the most crucial variable [27,36,37,38,39]. However, the social context (parents, friends, and teachers) also can act as a support for adolescents to practice physical activity [40]. Adolescents’ perceptions of their parents’ attitudes and behaviors in the area of physical activity are associated with self-perception of ability, attitudes, and attraction to physical activity or sports [41]. The subjective norm is the component of the TPB that measures this social context. However, some researchers have found that the perception that people from their environment see physical activity practice as something desirable, is the weakest predictor of TPB [26,39,42], and sometimes it was not significant [27,43].

Although various studies support the validity of the TPB in predicting physical activity, some research points to the clear conclusion that the TPB does consider all the determinants of intentions and behavior [26,44]. For these reasons, many researchers propose modifications to these theories. This idea is supported by [29], who postulates that the TPB is open to additional predictors if they show that they capture a significant portion of behavioral intentions after the current variables of the theory have been considered.

For this reason, the role of self in understanding exercise behavior has received increased attention during the last years [45]. Hence, self-related variables have been studied to understand exercise adherence [46]. This is the case of the physical self-concept and the exercise or athletic identity. The relationship between exercise adherence and physical self-concept, has been studied for some decades [47]. Several studies has demonstrated, direct relationships of physical self-concept with physical-sport practice [48,49,50,51]. The physical self-concept is defined as the set of physical self-perceptions that is structured hierarchically in four dimensions corresponding to the self-perceptions of physical ability, physical condition, strength, and physical attractiveness [38].

The relationship between intention and being active seems to be influenced by the subjects’ perception of their physical self-concept [52]. A high physical self-concept predicts a high level of physical activity in leisure time [53]. Moreover, the perception of sports attractiveness and competition, but not strength or fitness, predict the greater involvement in physical activity [50]. Along these lines, some studies highlight that physical condition was the dimension of physical self-concept with slightly higher values than the other dimensions of this, having a high predictive value in the practice of physical activity [41,54]. However, other research presents little relationship between the perception of physical attraction and the practice of physical activity, being excluded this variable in most cases [55]. About the analysis of physical self-concept in terms of gender, various studies have shown that women tend to have a lower assessment than men, and the dimensions related to physical-sports practice also differ [55,56,57,58].

On the other hand, regarding the second self-related variable, identity theory proposes that identities and behaviors are congruent. Hence, a strongly perceived adjustment between identity and a particular behavior is related to a strong intention to represent that behavior, but also makes the realization of the behavior more likely [59]. Therefore, a general perception of physical competence and identity as a physically active person, could positively influence the development of physical activity intentions and behavior during leisure time [60]. Studies on physical activity have shown the importance of identity in predicting physical activity intentions after the components of the TPB have been considered [43,61,62,63]. In the same vein, several studies have found that athletic identity increased the predictive value of the intention, after the components of TPB have been considered [64,65].

**Figure 1:**
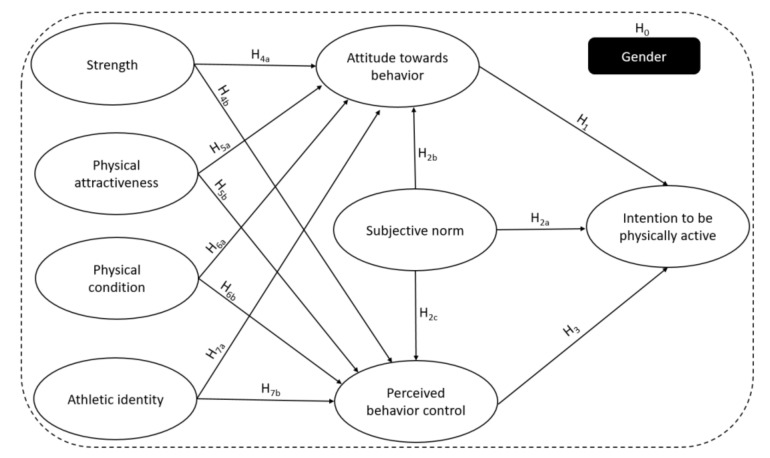
Model of intention to be physically active after finishing secondary school with gender as a moderator.

However, in the literature of social psychology, the process by which identity influences intention and behavior is still understood. Most studies have demonstrated small to large effects of identity on intention [44,66],. and identity is strongly correlated with attitudes [62,66], and perceived behavioral control [44,65]. Moreover, some researchers have found self-identity to be the second most reliable predictor of the intention to be physically active [32,43,67]. However, few other studies have examined the role of self-identity in the antecedents of TPB in human behavioral intentions [68]. Besides, physical self-concept [49] and athletic identity [43], are also known to dictate people’s physical activity awareness, no studies have included both of them within the TPB framework to analyze the moderating effect of gender. Hence, this study aims to analyse if there are significant differences in the variables related to the intention to be physically active after graduation according the adolescents’ gender. Moreover, it aims to analyze if the adolescents´ gender has a moderating effect on the antecedents of the intention to be physically active after graduation by using the TPB framework, and integrating the physical self-concept dimensions and athletic identity variables. For this purpose, the following hypotheses are enunciated which can be seen summarized in Figure 1.

Gender hypothesis:

− H_0_: Gender exerts a moderating effect among all the variables of the model, explaining the intention to be physically active after graduation.
− TPB’s variables hypotheses:
− H_1_: Attitude towards physical activity practice directly and positively influences the intention to be physically active after graduation.
− H_2a_: The perception that people from their environment see physical activity practice as something desirable (subjective norm), directly and positively influences the intention to be physically active after graduation.
− H_2b_: The perception that people from their environment see physical activity practice as something desirable (subjective norm) directly and positively influences the attitude towards physical activity practice.
− H_2c_: The perception that people from their environment see physical activity practice as something desirable (subjective norm), directly and positively influences the perceived behavioral control.
− H_3_: The perception of having the control and capacity to practice physical activity regularly (perceived behavioral control), directly and positively influences the intention to be physically active after graduation.
− Self-concept dimensions’ hypotheses:
− H_4a_: The strength perception directly and positively influences the attitude towards physical activity practice.
− H_4b_: The strength perception directly and positively influences the perception of having the control and capacity to practice physical activity regularly (perceived behavioral control).
− H_5a_: The physical attractiveness perception directly and positively influences the attitude towards physical activity practice.
− H_5b_: The physical attractiveness perception directly and positively influences the perception of having the control and capacity to practice physical activity regularly (perceived behavioral control).
− H_6a_: The physical condition perception directly and positively influences the attitude towards physical activity practice.
− H_6b_: The physical condition perception directly and positively influences the perception of having the control and capacity to practice physical activity regularly (perceived behavioral control).
− H_7a_: The athletic identity directly and positively influences the attitude towards physical activity practice.
− H_7b_: The athletic identity directly and positively influences the perception of having the control and capacity to practice physical activity regularly (perceived behavioral control).

This study is significant for both theory and practice. Theoretically, it demonstrates the possibility to extend the TPB with two more variables: physical self-concept dimensions and athletic identity. Moreover, it analyses the moderating effect of gender on these variables related to physical activity practice. From the practical perspective, the results inform local health authorities and sport politicians on ways to promote physical activity practice for adolescents in general, and especially for female adolescents that are more necessaries. These findings could help to create policies to decrease the gender gap in the physical activity practice, and increase the physical activity levels of female adolescents. This fact is important because there is an urgent need to promote and retain girls’ participation in physical activity [6].

## 2. Materials and Methods

### 2.1. Participants

The target population of this study is students in the 4th course of secondary education and A levels of a high school located in Valencia (Spain). A levels refer to the last stage of secondary education in Spain, is voluntary, and it lasts two years (normally 16–18 years). A non-probabilistic sampling was used for this, being this intentional or of convenience. The sample is comprised of 256 students who were between 16 and 19 years old, with an average age of 16.58 years old (SD = ± 0.61). The 94.90% of students were under 18. The 55.50% of the students were male (n = 114), while 44.50% were female (n = 142). According to the courses, 46.80% were students in the 4th grade of secondary school, 47.10% were students in 1st grade of A levels, and 6.10% were students in 2nd grade of A levels.

### 2.2. Instrument

For the data collection, a questionnaire composed of 31 items was created, divided into different areas, taking the following scales as a reference:

(a) The measure of the Intentionality to be Physically Active (MIFA) from [69] translated into Spanish and validated by [16]. This scale measures the personal intention to be physically active after graduating. It consists of five items grouped into the same factor or variable, so they measure the same aspect (i.e., “I am interested in the development of my physical form”). The items are preceded by the phrase “Regarding your intention to practice some physical-sports activity
(b) The scale of Physical Self-Concept (CAF-A) extracted from [70], which validated the psychometric properties of this Spanish version with adolescents. This scale consists of eight items that measure four dimensions of physical self-concept: strength (two items: “I have more strength than most people my age “and “I am strong”); physical attractiveness (two items: “I feel happy with my body image” and “I like my face and my body”); physical skills (two items: “I’m one of those people who has trouble learning a new sport” and “I see myself clumsy in sports activities”); and physical condition (two items: “I have a lot of physical endurance” and “I can run and exercise for a long time without getting tired”). For this purpose, an ascending Likert scale is used, where 1 means false, 2 means almost always false, 3 means sometimes true sometimes false, 4 almost always true and 5 true.
(c) The scale of the Theory of Planned Behavior by [71], in which four items compose the subjective norm construct, seven for the perceived behavioral and the attitude towards the behavior. Subjective norm, refers to the assessment that people in their immediate environment make of physical and sporting practice (i.e., “Most of the people who are important to me think that I should do sport or physical exercise at least three times a week”). Specifically, the items refer to what people in the immediate environment think (one item), want (one item), and expect (one item) about doing sport or physical exercise at least three times a week. Perceived behavioral control measures the subjects’ perception of their ability to perform physical exercise or sport at least three times a week (i.e., “I think I can do sport/exercise at least three times a week”). Precisely, it measures predisposition (one item), the locus of control (one item), capacity (one item), and finally, difficulties (one item). Attitude towards behavior measures how important is to practice physical activity three times a week, by presenting different adjectives: important (one item), enjoyable (one item), relaxing (one item), useful (one item), beneficial (one item) to intelligent (one item). As a measure of these items, a 5-point ascending Likert scale is used, where 1 means no agreement and 5 means total agreement.
(d) Exercise Identity Scale (EIS) by [72], in its Spanish version by [73]. The scale is composed of five items that measure the subjects’ perception of their sports identity (i.e., “I am the type of person who enjoys exercising/sports in my free time”). The items refer to whether they perceive themselves as an athletic person. Also, they refer to the feeling of discomfort if they were forced to stop practicing physical activity and the enjoyment of doing physical activity or sport during free time. To measure these items a 5-point ascending Likert scale is used where 1 meant completely disagree, and 5 completely agree. Finally, several sociodemographic variables that are considered of interest for the study are measured, such as: the course, age, and gender of the students.

### 2.3. Procedure

A quantitative research with a cross-sectional design was adopted in this study. The questionnaires were administered to as many 4th course students of secondary education and A levels students as possible, from this specific high school. It was decided to opt for this group of students because they are those who are close to finishing their compulsory schooling or their period of schooling in high school. Hence, they will finish the subject of Physical Education, and for some of them, this may mean stopping the only physical activity they practice.

Ethical approval was obtained from the Ethics and Human Research Committee of the University of Valencia. This questionnaire was sent to the headteacher for review and consent to administer it to the students. Once the principal gave her consent and informed the physical education teacher, the questionnaires were administered to the students before the physical education classes began. Throughout the process, students were informed of the anonymous nature of the questionnaire and the voluntary nature of completing it. The principles of the Declaration of Helsinki [74] were followed for this type of research.

As for the time required to answer the questionnaire, this was approximately 10–15 min, and there were no incidents due to delivery delays.

### 2.4. Data Analysis

The data are analyzed using the SPSS statistical package (Version 23, IBM Corp, Armonk, NY, USA) and PLS 3.0 (SmartPLS GmbH, Bönningstedt, Germany). Descriptive and correlation analyses and the Student t-test are performed with SPSS using the partial least squares (PLS) program, structural modeling techniques are performed to test the hypotheses. Given the characteristics of the proposed model, structural equation techniques are used to test the hypotheses. In particular, a multivariate analysis based on Partial Least Squares is used. When exploratory studies are carried out, and relatively small samples are used, this multivariate statistical technique is more appropriate than others, based on covariance analysis [75]. PLS analysis provides results for both the structural model (casual relationships) and the measurement model (reliability and validity of indicators) [76].

Also, to explore possible differences in results between the two countries, a multi-group analysis has been performed. This technique looks for statistically significant differences in trajectory coefficients between sub-samples [77]. However, previously it was necessary to assess measurement invariance. To do so, we rely on a three-step procedure to analyze the measurement invariance of the composite model (MICOM), which is very appropriate when using variance-based SEM, such as partial least squares (PLS) path modeling [78]. The three-steps that MICOM procedure are [78]: (1) configural invariance (equal parameterization and way of estimation), (2) compositional invariance (equal indicator weights), and (3) the equality of composite mean values and variances. If configural and compositional invariance is established, partial measurement invariance is confirmed. This step allows one to compare the path coefficient estimates across the different samples. Hence, in this study, only the two first steps were performed.

Finally, to evaluate the fit of the model SRMR, d_ULS, and d_G indexes. Also the SRMR index is used, because is considered as a goodness of fit measure for PLS-SEM that can be used to avoid model misspecification [79]. According to [80], SRMR should be lower than 0.08 to be considered a good fit model. Moreover, [81], recommends using d_ULS (i.e., the squared Euclidean distance) and d_G (i.e., the geodesic distance), which are two different ways to compute this discrepancy. For the exact fit criteria (d_ULS and d_G), their original value is compared against the confidence interval created from the sampling distribution. The confidence interval should include the original value, which means not to be statistically significant. Hence, the upper bound of the confidence interval (95% or 97%) should be larger than the original value of the exact d_ULS and d_G fit criteria to assess that the model has a good fit.

## 3. Results

This section presents the statistical analyses to respond to the objectives and hypotheses raised in this study. Firstly, the analyses of the reliability and validity of the scale are presented. After that, comparisons of means between groups, and finally, the results of the multi-group analysis.

### 3.1. Reliability and Validity Analysis

#### 3.1.1. Convergent Validity and Reliability of Measurements

Firstly, the convergent validity and reliability of the measures are analyzed according students’ gender. Convergent validity is determined through the statistical significance of the factorial loads of the indicators of each latent construct. Table 1 shows that all standardized loads (λ) are higher than 0.60 [82]. For the sub-samples, according to the gender of the students, this criterion is met. Therefore, there is a convergent validity of the different items that make up the variables under study.

Regarding reliability, this is calculated through composite reliability (CR) and Cronbach’s alpha. The minimum value considered adequate is 0.70 [83], which in all cases is appropriated, except in the dimension of physical self-concept called ability (see Table 1). Finally, the mean extracted variance (AVE) reflects the total amount of indicator variance collected by the latent variable. Generally, AVE value should be higher than 0.50 [70]. As can be observed in Table 1, all constructs present an AVE higher than 0.50, except for the dimension of physical self-concept called physical skill. Therefore, this dimension is eliminated to make the model.

#### 3.1.2. Discriminant Validity

Discriminant validity is then analyzed for each of the sub-samples. To evaluate the presence of discriminant validity between constructs, the square root of AVE must meet the criterion of being superior to the correlation between constructs [83]. Table 2 shows the correlations between constructs and, diagonally, the square root of AVE. If the results of the table are observed, it can be proved that the established criterion is met. Therefore, there is a discriminating validity between the constructs.

Also, physical attractiveness, physical condition and strength, subjective norm, and athletic idiosyncrasy were significantly correlated with the variable attitude towards behavior and perceived behavioral control (*p* < 0.05) in both subsamples (male/female). Also, attitude towards behavior perceived behavioral control, and the subjective norm was significantly correlated with intention to be physically active after graduation (*p* < 0.05) in both subsamples.

### 3.2. Mean Comparisons Between Adolescents Gender

The averages of these variables are compared in terms of adolescents’ gender. In all cases, the means were higher for boys than for girls. Moreover, there are statistically significant differences in the variables’ attractiveness (*p* = 0.031), condition (*p* < 0.001), and strength (*p* <0.001). Statistically, significant differences are also in the variables, athletic identity (*p* < 0.001), and intention to be physically active (*p* < 0.001). These results are in Table 3.

### 3.3. Invariance Assessment

Two steps are followed to ensure partial measurement invariance to proceed with the multi-group analysis. First, the configurable invariance is checked. In this case, due to the model settings in the previous step of this analysis, the configurations of the PLS route models are the same for both samples (male and female). This is a necessary prerequisite for setting the configuration invariance in step 1 of the MICOM procedure. Also, because group-specific model estimates are also based on the same algorithms, configurable invariance was ensured.

Secondly, it is checked whether the composite scores are the same in all groups despite possible differences in weights to ensure compositional invariance. For this purpose, the permutation procedure is applied with 500 permutations and a significance level of 5% for each combination of the sample (male adolescents and female adolescents). Subsequently, the correlations of the original composite score (*c)* are compared with the empirical distribution of the correlations of the composite score resulting from the permutation procedure (*c_u_*). If it is equal to or higher than the 5% *c_u_* quantile, the compositional invariance is established [84]. The results in Table 4 show that this criterion is met in the comparison between the samples by the genus. Therefore, a partial measurement invariance between these two groups is ensured, which allows the multi-group analysis to be performed.

### 3.4. Structural Equation Models: A Multi-Group Analysis

Once the compositional invariance is checked, the multi-group analysis is performed. The multi-group analysis compares the trajectory coefficients between the samples according to the gender of the adolescent to identify significant differences [71]. The same model is previously calculated for each of the sub-samples. In Table 5 these results are presented.

First, the model for male adolescents presents a good fit index, as the SRMR (.06) was lower than 0.08 [80]. Also, both the values of d_ULS with 1.60 (HI_95_ = 2.04) and d_G with 0.93 (HI_95_ = 1.19), were found within the confidence interval, being thus not statistically significant values (*p* > 0.05), as recommended by the literature [81]. About the predictor variables, the variables physical attractiveness (β = 0.36; *p* < 0.001), physical condition (β = −0.24; *p* < 0.05), and athletic identity (β = 0.56; *p* < 0.001), exerted a direct influence on the control of perceived behavior. Physical attractiveness and athletic identity did it positively, while physical condition did it in a negatively, explaining 42% (R^2^ = 42) of the variance of this variable. Therefore, H_5b_ and H_7b_ hypotheses were accepted. However, the H_2c_, H_4b_, and H_6b_ hypotheses were reputed. These results may be because the condition did exert a direct influence in a statistically significant way, but negatively, and the other two variables did not turn out to have a statistically significant influence. Also, athletic identity directly and positively influenced attitude towards behavior (β = 0.37; *p* < 0.001), explaining 20% (R^2^ = 0.20) of the variance. Therefore, only the H_7a_ hypothesis was verified. However, the hypotheses H_2b_, H_4a_, H_5a_, and H_6a_ are refuted. These hypotheses are refuted because none of the other variables proved to influence in a statistically significant way. Finally, there are also statistically significant relationships between the attitude towards behavior (β = 0.21; *p* < 0.001), the perceived behavioral control (β = 0.28; *p* < 0.001), and the subjective norm (β = 0.36; *p* < 0.001), with the intention to be physically active. The relationships between these variables are directly and positively. Therefore, the hypotheses H_1_, H_2a_, and H_3_ were accepted. Thus, the model was able to explain 38% (R^2^ = 0.38) of the intention to be physically active after graduating.

Second, the model for female adolescents also showed a good fit index, as the SRMR (0.06) was lower than 0.08 [80]. Moreover, both the values of d_ULS with 1.62 (HI_95_ = 2.19) and d_G with 1.24; (HI_95_ = 1.66), were found within the confidence interval, being thus not statistically significant values (*p* > 0.05), as recommended by the literature [81]. Regarding the predictor variables, the variables physical condition (β = 0.24; *p* < 0.01), and strength (β = −0.24; *p* < 0.001), as well as athletic identity (β = 0.56; *p* < 0.001), exerted a direct influence on the perceived behavioral control. Therefore, H_6b_ and H_7b_ hypotheses were accepted. However, the H_2c_, H_4_, and H_5b_ hypotheses, which proposed that the subjective norm, strength, and physical attractiveness exerted a direct and positive influence on the perceived behavioral control, were reputed. The subjective norm and attractiveness did not do so significantly, while force did so in a statistically significant but negative way. Thus, it was possible to explain 51% (R^2^ = 51) of the variance in perceived behavioral control. Also, athletic identity directly and positively influenced the attitude towards behavior (β = 0.56; *p* < 0.001), explaining 30% (R^2^ = 0.20) of the variance. Therefore, only the H_7a_ hypothesis was verified. However, the hypotheses H_2b_, H_4a_, H_5a_, and H_6a_ are refuted. Finally, the variables attitude towards behavior (β = 0.28; *p* < 0.001), perceived behavioral control (β = 0.44; *p* < 0.001), were found to directly and positively influence the intention to be physically active after the graduation. Therefore, hypotheses H_1_ and H_3_ are accepted. However, hypothesis H_2a_ is refuted, since the relationship between SN and IPA was not statistically significant. Thus, the model was able to explain 38% (R^2^ = 0.38) of the intention to be physically active after graduation.

**Figure 2:**
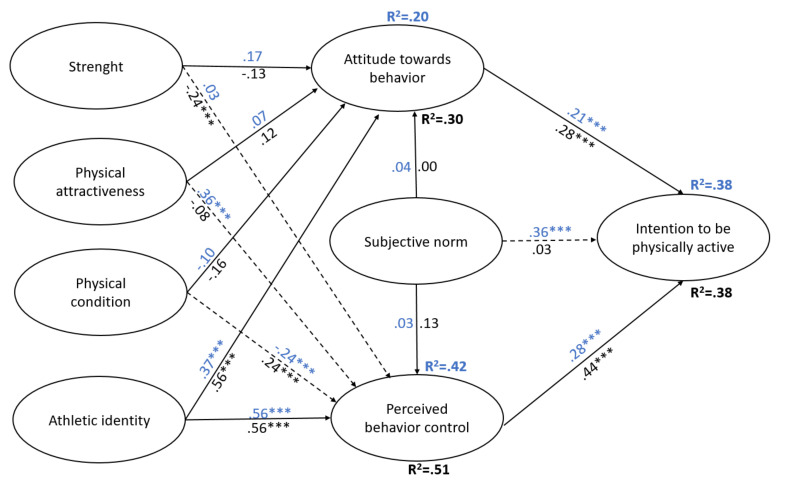
Model of intention to be physically active after graduating with male vs. female sample. Note: male coefficients are below the line in blue, and female coefficients are under the line in black. Discontinuous lines refer to significant differences between male/female path coefficients.

Finally, this statistical analysis aims at detecting statistically significant variations in the sub-samples according to the gender of the adolescents (see Table 5). The variance’ percentage of the intention to be physically active after graduation explained by the models according to gender is the same for both (R^2^ = 0.38). As for the predictor variables, statistically, significant differences were found in the paths between the subjective norm and the intention to be physically active after graduation (*pc*male-*pc*female = 0.33; *p* < 0.01), is significant for men but not for women this coefficient. Also, statistically, significant differences were found between the three dimensions of physical self-concept (physical attractiveness, physical condition, and strength) and perceived behavioral control (*pc*fmale - *pc*fmale = 0.44; −0.48 *p* < 0.001; 0.27, *p* < 0.05), respectively. Hence, hypothesis H_0_, was partially supported. These results are presented graphically in Figure 2.

## 4. Discussion

During the adolescent stage is when the highest rate of abandonment of physical-sports practice occurs. Moreover, the dropout rate is much higher in women than in men [6]. Therefore, this research aimed to analyze whether there were significant gender differences in the variables related to the intention to practice physical activity after graduating; and whether there is a moderating effect of gender on the variables that predict the intention to practice physical activity after graduation.

The results of this study found that, in all the variables related to physical-sports practice, it was male adolescents who presented higher averages than those of the female gender. Specifically, these differences were statistically significant in the case of all the dimensions of physical self-concept analyzed, in the athletic identity, and the intention to be physically active at the end of schooling. These results are in line with several studies that have found that women usually present lower averages in physical self-concept than men [56,58,84,85]. This finding is in line with [55], who points out that the most significant differences in physical self-concept between genders are found in the adolescent stage. However, they are against others researches [86] who show that girls have a more favorable perception of their physical appearance and physical strength than boys.

Several studies have also found statistically significant differences in the intention to be physically active according to gender, with men showing higher scores than women [19,87]. Therefore, these lower scores in the perceptions of variables related to physical activity practice can explain these existing differences in physical activity levels according to gender. This result may be due to cultural factors associated with gender since, in some countries where resources have been invested to achieve gender equity, girls practice more physical activity than boys [22]. Therefore, these results highlight the importance and necessity for specific interventions focused on increasing the physical-sports practice of female adolescents [6].

Concerning the predictive variables of the intention to be physically active, the results show that they are differences according to the adolescents’ gender. Firstly, for the male adolescents´ model, the perceptions of their physical attractiveness exert a direct and positive relationship in the perception of their capacity to practice physical activity continuously. This finding is in line with [48], who found that physical appearance is strongly associated with physical activity. However, in the case of female adolescences, physical attractiveness does not exert an influence on perceived behavioral control. These results are also in line with [10,75], who showed that physical attractiveness is positively related to physical activity in the boys, and negatively in the girls. These results could suggest that boys who perceive themselves with a good body image opt to engage in physical activity to maintain their good physical appearance [10,41]. However, the girls who are satisfied with their appearance, are probably less concerned about practicing physical activity

On the other hand, the results of this study show that, in male adolescents, the perception of their physical condition exerts a direct but negative relationship on the perception of their capacity to practice physical activity regularly. This result may be since usually male adolescents tend to present high scores in physical condition compared to girls [55,57]. Therefore, only if they perceive that they have a low physical condition, they feel more motivated or capable of performing such physical sports practice continuously. Nevertheless, this is just one possible explanation of the results obtained. However, in the case of the female adolescents, the perception about their physical condition exert a direct and positive relationship with the perception of their capacity to practice physical activity regularly. These results are in line with those found by [75], who shows that only the subscale of physical self-concept is related to the physical activity practice in the case of women. These results are in line with [50], who highlights that the reduction of physical activity among girls who are advanced in their state of maturity could be counteracted through work on improving physical self-concept (perception of physical condition). Therefore, physical education classes should develop activities that allow adolescents to improve their physical condition, such as aerobic activities. They will develop the perception of their capacity to practice physical activity regularly, and therefore, their intention to be physically active.

Regarding the relationship between the strength perception and the perception of their capacity to practice physical activity regularly, this relationship was not significant in the case of male adolescents. However, it was statistically significant in the case of female adolescents, but exerting a negative relationship. It may be since the activities that girls prefer to do in their free time are more related to physical condition and not with force. In fact, among the reasons why girls tend to practice physical-sports activity, they stand out for being healthy and fit [88]. Therefore, improving the perception of their strength would not generate higher levels of physical activity practice in adolescents regardless of their gender.

On the other hand, in the case of both boys and girls, the athletic identity exerts a positive and direct influence both through the perception of control to practice physical activity regularly, as well as the attitude towards this regularly physical activity practice. Therefore, these results are in line with several studies that highlight the importance of identity in predicting intentions in general [64], and mainly intentions to practice physical activity [43,62,63]. It implies that abandonment or lack of or little practice of physical activity in adolescents is something that usually lasts into adulthood [76]. Hence, the importance of generating such habits of extra-curricular physical activity practice during the schooling period. This finding implies that when health, education, or sports authorities implement intervention programs to promote physical activity practice, they should try to construct new identities. Creating new identities for the practice of physical activity means being able to make those adolescents perceive themselves as people who practice a physical sport regularly, and who consider such behavior as an essential dimension of their reason for being. In that way, the meanings, expectations, and activities associated with becoming a particular type of person should be incorporated into the self to complete the behavioral change process [64].To this end, social identity theory offers several recommendations to improve the athletic or exercise self-identity of the persons, such as the following [76]: offer opportunities to adopt the role of being a physically active person, that means, to give them the opportunity to practice physical activity regularly (e.g., to engage them in physical activities at less three times per week); reward the evidence of adoption of this role (e.g., receiving a reward or recognition, such as extra points for participating in sports activities outside of PE classes); being seen by others who are essential in adopting the role and obtaining evidence of reward for it (e.g., organizing sports races or tournaments from the PE area where parents and friends are invited); and finally, acting as a role model to persuade others of the importance of this role (e.g., receive a talk of a famous sportsman or woman about the benefits of physical activity)

Finally, in the case of both male and female adolescents, the favorable attitude towards physical sports practice and the perception of having the control and capacity to practice physical activity regularly, proved to have a direct and positive influence on the intention to be physically active after graduation. Of these two variables, the perception of having the control and capacity to practice physical activity regularly was the most influential variable in the case of girls. This finding is in line with previous studies, which have also shown that control of perceived behavior is the most influential variable in physical sports practice intentions [36,37,43]. However, perception that people from their environment see physical activity practice as something desirable only turned out to influence the intention of physical-sports practice in the case of boys. Furthermore, it was the most crucial variable in the case of boys, even though numerous studies have found no relationship or very weak links. These results show that the physical-sports practice of adolescents is linked to the lifestyles of their social group of reference (family, peer group, and school) [76]. Furthermore, it should be borne in mind that as age advances, the group of friends becomes the most influential factor. Therefore, to promote healthy habits in adolescents, it appears necessary to carry out an action plan that involves families, schools, and public administrations [85].

The results of this study highlight the moderating effect of gender in explaining intentions to be physically active after graduation. Specifically, there are significant differences in the paths between the perception that people from their environment see physical activity practice as something desirable and the intention to be physically active after graduation, being significant for men but not for women this coefficient. It may be because, in the case of girls, participation in physical activity is based more on personal motivation, and less on the influence or pressure of others in the environment [89]. Also, another possible explanation is that girls have a higher internal locus of control than boys, and therefore, the opinion of the people around them is not as influential. It means that adolescent girls perceive that behaviors or events occur primarily as an effect of their actions, and give less importance to what those around them think. In other words, they perceive that they have control of their lives. Also, significant differences are found between the three dimensions of physical self-concept (strength, physical attractiveness, and physical condition) and the perception of having the control and capacity to practice physical activity regularly. These results are in line with [48], who highlight that sex is a significant moderator for general physical self-concept.

To conclude, educational interventions that have an impact on the creation of healthy lifestyles, from the perspective of the practice of physical activity are necessary in young people [90]. To this end, the school environment offers multiple possibilities for encouraging the regular practice of physical activity. It constitutes one of the most effective places for changing harmful lifestyles and promoting overall health [91]. As a central field of physical activity, experiences in Physical Education (PE) classes can stimulate the development of students’ general perceptions of physical competence and athletic identity [63,92,93]. Hence quality PE supports children to develop behavior patterns that keep them physically active throughout their lives [94,95]. Therefore, the PE teacher has a vital role in increasing future practice intent and should adopt motivational strategies that help change adolescent behavior [96].

### Limitations and Future Lines of Research

The present study has some limitations which should be considered in future research, one of which is the limited size of our sample. This number should be increased to establish more robust statistical conclusions and to generalize these results to the entire population of female and male adolescents. Also, it would be interesting to compare the variables that predict the intention to be physically active of adolescents after graduation according to gender, in students from countries with different rates of physical inactivity levels. Furthermore, it is a transversal study, so it has not been possible to follow the evolution of the physical practice of the sport. Therefore, it would be interesting to analyze how the variables predicting the intention to practice physical activity after graduation varied from the time students start secondary education until they finish it. Moreover, the intention-behavior gap is widely recognized as a limitation in the health behavior research community. Hence future research lines should analyze the link intention-behavior, by providing more objective measures that could be by measuring physical activity levels with accelerometers. Finally, this is a non-experimental study. Therefore, in future studies, it would be interesting to carry out specific interventions according to the adolescents’ gender, based on the results obtained in this study, to know if the intentions of physical activity practice increase in both male and female adolescents.

## 5. Conclusions

The data from this study show that male adolescents present higher averages in all variables related to physical activity practice. It was found that gender moderates the relationships between particular variables related to physical-sports practice intentions at the end of secondary school. Therefore, when designing educational policies and physical education classes for the promotion of physical activity practice among adolescents, gender is a variable that must be considered. Moreover, these policies for the promotion of physical-sports practice are more important in the case of adolescent girls than boys.

In the case of male adolescents, policies in the area of sports should be mainly oriented towards improving their perception to feel capable of practicing physical activity of at least 60 minutes three days a week. A positive attitude towards physical and sports practice should also be encouraged. Likewise, the perception that people from their environment (parents, friends, and classmates) see physical activity practice as something desirable, is also important. Working on athletic identity, i.e., to perceive themselves as a sporty person, also helps improve their attitude towards physical activity and their perception of having the control and capacity to practice physical activity regularly. Finally, improving the perception of their physical attractiveness can help to generate higher levels of intentions to be physically active, indirectly.

As regards sports policies to encourage physical activity among female adolescents, the primary variable to develop should be the positive attitude towards physical activity. In other words, they should consider the continued practice of physical sport as something good and positive for their health. Likewise, the fact perception of having the control and capacity to practice physical activity regularly for at least 60 minutes three days per week, is also an aspect that can help to create this adherence. Besides, the generation of an athletic identity is critical, that is, that they perceive themselves as sportsmen or women. Finally, the improvement of the perception of their physical condition is also an essential aspect for the generation of this intention to be physically active after graduation.

## 6. Practical Application

The results of this study contribute to the literature on how to encourage physical activity practice in adolescents considering gender. The results show the necessity to adapt the measures to promote physical activity practice at this stage, according to the adolescents’ gender. Therefore, from PE classes, these considerations should be considered to achieve higher levels of physical-sports adherence in girls.

Regardless of the gender of the adolescents, physical education classes should include activities that improve adolescents’ perception of having the control and capacity to practice physical activity at least three times a week. Also, a positive attitude towards physical sports practice should be encouraged on an ongoing basis, and students should be perceived as physically active (athletic identity). To this end, famous sportsmen and women could be brought into the classroom to tell the students about the benefits of physical sports practice. Moreover, students could do essays in which they would have to search for the benefits that the practice of physical-sports activity reports in a continuous way for health, as well as which are the most appropriate levels of these. Another possible activity for students would be to download an application to their mobile phones, in which they would count the steps taken every day and establish weekly objectives to be achieved. The teacher, based on this, could weekly or monthly, proclaim the role of the most active student in each class. Likewise, they could keep a diary of the physical-sports practice they do every week, and try to set themselves some more and more demanding objectives every week to reach the recommended levels of physical activity. Also, a “contract” could be established at the beginning of the course with the PE teacher to maintain or increase the physical activity level. At the end of the course, if the target is fulfilled, it could be reflected in the students’ marks (incentives). Also, the teacher should encourage the participation of students in local sports events (e.g., half a point more for each participant in a sports event).

However, in the case of women, in addition to the activities outlined above, PE teachers should encourage girls to engage in activities that allow them to improve their physical condition. These activities include aerobic or endurance-type activities such as running, dancing, cycling, swimming... or any type of activity that requires repeated practice. Therefore, aerobic activities, such as games that require running and moving continuously, should be performed in PE classes. Besides, they should make students aware that these activities improve their physical condition, and they should be aware if throughout the course, they feel they have improved their physical condition. Therefore, a test that measures physical condition at the beginning, middle, and end of the course should be used. Also, they should be encouraged to practice these types of activities outside the PE classes, and to be aware of the progress in their physical condition. However, they should avoid activities to improve strength, as this may be counterproductive in raising their levels of physical activity adherence.

Finally, in the case of boys, in addition to the activities proposed at the beginning of this section, PE teachers should carry out activities that involve the adolescents’ immediate environment, and that improve the perception of their physical attractiveness. To this end, the PE area can organize sports days involving the students’ families, in which the benefits of physical-sports practice and the appropriate levels of it are presented. Moreover, these activities should encourage physical and sports practice within the family. Finally, in PE classes, students should be encouraged to monitor the changes that occur in their bodies when doing physical sports. In this way, they could be aware not only of the improvements produced at a physiological and psychological level but also at a physical level. However, they should avoid activities to improve the perception of their physical condition, as it seems that this may be negative for their physical activity adherence.

## Figures and Tables

**Table 1:** Indicators, loadings (λ), alpha de Cronbach (α), Compose Reliability (CR), and AVE of the total, male, and female samples.

Constructs	Indicators	Male Sample					Female Sample			
		λ	λ	α	CR	AVE	λ	α	CR	AVE
**ATB**	**1**	0.78	0.80	0.90	0.92	0.66	0.76	0.82	0.87	0.52
	**2**	0.77	0.77				0.76			
	**3**	0.71	0.76				0.64			
	**4**	0.81	0.85				0.77			
	**5**	0.78	0.84				0.67			
	**6**	0.81	0.86				0.73			
**PATR**	**7**	0.92	0.89	0.76	0.89	0.81	0.91	0.81	0.91	0.84
	**8**	0.89	0.90				0.92			
**CON**	**9**	0.94	0.92	0.85	0.93	0.87	0.94	0.87	0.94	0.88
	**10**	0.94	0.94				0.94			
**STR**	**11**	0.95	0.92	0.82	0.92	0.85	0.96	0.90	0.95	0.91
	**12**	0.94	0.92				0.95			
**PS**	**13**	0.83	0.78	0.41	0.41	0.77	0.82	0.58	0.59	0.83
	**14**	0.81	0.81				0.86			
**AI**	**15**	**0.89**	**0.89**	**0.89**	**0.92**	**0.70**	**0.87**	**0.92**	**0.94**	**0.76**
	**16**	0.71	0.62				0.83			
	**17**	0.82	0.82				0.82			
	**18**	0.92	0.91				0.92			
	**19**	0.92	0.93				0.91			
**CCP**	**20**	0.81	0.76	0.75	0.84	0.56	0.86	0.88	0.92	0.74
	**21**	0.73	0.62				0.80			
	**22**	0.82	0.85				0.88			
	**23**	0.92	0.75				0.89			
**IPA**	**24**	0.92	0.69	0.84	0.89	0.62	0.60	0.80	0.86	0.57
	**25**	0.64	0.81				0.89			
	**26**	0.85	0.73				0.60			
	**27**	0.67	0.82				0.79			
	**28**	0.81	0.86				0.79			
**SN**	**29**	0.71	0.61	0.74	0.74	0.51	0.85	0.81	0.88	0.71
	**30**	0.72	0.60				0.83			
	**31**	0.91	0.96				0.85			

Note: ATB- Attitude towards behavior; PATR-Physical attractiveness; PH- Physical condition; STR-Strength; PS-Physical skills; AI-Athletic identity; PBC- Perceived behavioral control; IPA-Intention to be physically active after graduation; SN-Subjective norm.

**Table 2:** Means comparisons variables related to physical activity intentions after finishing secondary school female vs. male sample.

	ATB	PATR	PC	STR	AI	PBC	IPA	SN
**ATB**	**0.81(0.71)**		.					
**PATR**	0.22**(0.22*)	**0.90 (0.92)**						
**PC**	22**(0.22*)	46***(0.37***)	**0.93(0.94)**					
**STR**	23**(0.21*)	0.38***(0.20)	39***(0.49***)	**0.92(0.96)**				
**AI**	39***(0.49***)	26**(0.13^*^)	52***(0.56***)	0.48***(0.41***)	**0.84(0.87)**			
**PBC**	14(0.26**)	28***(14*)	17*(0.37***)	0.79***(0.13**)	37**(0.53***)*	**0.75(0.86)**		
**IPA**	34***(0.39***)	28***(0.07)	0.57***(0.44***)	0.35***(0.35***)	18*(0.81***)	0.30***(0.44***)	**0.79(0.75)**	
**SN**	18*(0.26**)	08(0.08)	22**(0.19*)	0.49***(0.26**)	26**(0.41***)	39***(0.30**)	26***(0.29***)	**0.81(0.84)**

Note: * *p* < 0.05; ** *p* < 0.01; *** *p* < 0.001; ATB- Attitude towards behavior; PATR-Physical attractiveness; PH- Physical condition; STR-Strength; AI-Athletic identity; PBC- Perceived behavioral control; IPA-Intention to be physically active after graduation; SN-Subjective norm. In the diagonal in bold are the values of the square root of AVE. Male values are, on the left, and female values are on the right in brackets.

**Table 3:** Means comparisons of variables related to physical activity intentions after finishing secondary school female vs. male sample.

Variable	Male	Female	*p*	Cohen’s d
Attitude towards behavior	4.14 (0.76)	4.12 (0.63)	0.194	-
Attractiveness	3.93(0.95)	3.65 (1.09)	0.031	0.27
Condition	3.82 (1.08)	3.06(1.11)	0.000	0.69
Strength	3.50 (0.98)	2.70 (1.18)	0.000	0.74
Athletic identity	4.21 (0.94)	3.72 (1.22)	0.000	0.45
Perceived behavioral control	4.52 (0.71)	4.39 (0.87)	0.194	-
Intention to be physically active	4.41 (0.75)	4.07 (0.78)	0.000	0.44
Subjective norm	3.83 (0.95)	3.67 (1.05)	0.728	-

**Table 4:** Compositional invariance assessment.

Variables	c	5% Quantile of *cu*
Attitude towards behavior	1.00	0.98
Attractiveness	1.00	0.95
Condition	1.00	0.99
Strength	1.00	0.99
Ability	0.99	0.62
Athletic Identity	1.00	1.00
Perceived behavioral control	0.99	0.99
Intention to be physically active	1.00	0.99
Subjective norm	0.94	0.87

**Table 5:** Multi-group analysis of the model to predict the intention to be physically active after graduation according to the gender: path coefficients, R^2^, and fit indexes of the model.

Path Coefficients (p_c_)	Male Students (t Value)	Female Students (t Value)	Male–Female	t Value
**ATB > IPA**	0.21***(2.70)	0.28***(2.90)	−0.07	0.58
**PATR > ATB**	0.07 (0.79)	0.12(1.03)	−0.05	0.30
**PATR > PBC**	0.36***(4.65)	−0.08(0.82)	0.44***	3.47
**PC ->ATB**	−0.10 (0.84)	−0.16 (1.54)	0.06	0.35
**PC ->PBC**	−0.24*(2.09)	0.24** (2.46)	−0.48***	3.02
**STR > ATB**	0.17(1.87)	0.13(1.36)	0.04	0.33
**STR >PBC**	0.03(0.37)	−0.24***(3.20)	0.27*	2.39
**ID >ATB**	0.37 ***(3.32)	0.56***(5.20)	−0.19	1.23
**ID>PBC**	0.56 ***(5.00)	0.56***(6.32)	0.00	0.02
**PBC>IPA**	0.28 ***(3.23)	0.44***(4.66)	−0.16	1.15
**SN>ATB**	0.04(0.36)	0.00(0.02)	0.03	0.23
**SN>PBC**	0.03 (.0.27)	0.13(1.19)	−0.10	0.68
**SN>IPA**	0.36***(4.49)	0.03(0.28)	0.33**	2.61
**R^2^**				
**ATB**	0.20***	0.30***		
**PBC**	0.42***	0.51***		
**IPA**	0.38***	0.38***		
**SRMR**	0.06	0.06		
**d_ULS**	1.60 (HI_95_ = 2.04)	1.62 (HI_95_ = 2.19)		
**d_G**	0.93 (HI_95_ = 1.19)	1.24 (HI_95_ = 1.66)		

Note: Attitude towards behavior; PATR-Physical attractiveness; PH- Physical condition; STR-Strength; AI-Athletic identity; PBC- Perceived behavioral control; IPA-Intention to be physically active after graduation; SN-Subjective norm.
